# Bacteria-related signals in brain metastases: evidence boundaries, tumor-microenvironment remodeling, and translational prospects

**DOI:** 10.3389/fcell.2026.1893882

**Published:** 2026-07-22

**Authors:** Guicheng Kuang, Zhenghaonan Qiu, Lingxiao Li, Jinjian Bai, Hang Ji, Yi Liu

**Affiliations:** 1 Department of Neurosurgery, West China Hospital, Sichuan University, Chengdu, Sichuan, China; 2 West China School of Medicine, Sichuan University, Chengdu, Sichuan, China

**Keywords:** bacterial signals, blood–tumor barrier, brain metastases, gut–brain–metastasis axis, intratumoral microbiota, low-biomass contamination, translational biomarkers, tumor microenvironment

## Abstract

Brain metastases (BrM) develop within a highly specialized central nervous system niche shaped by the blood–brain barrier/blood–tumor barrier, brain-resident stromal cells, myeloid populations, and distinct metabolic constraints. Emerging studies suggest that bacteria-related signals can be detected in primary and metastatic brain tumors; however, their biological meaning remains incompletely defined. In particular, low-biomass brain tissues are highly vulnerable to reagent contamination, environmental carry-over, batch effects, and bioinformatic misclassification, making it essential to distinguish molecular bacterial traces from viable intratumoral bacteria or a *bona fide* tumor microbiome. In this review, we propose a graded conceptual framework that separates bacterial signals/elements, intratumoral bacteria, and intratumoral microbiota/microbiome according to evidentiary strength. We summarize current evidence for the spatial and cellular localization of bacteria-related signals in BrM and discuss potential source models, including primary-tumor carry-over, hematogenous dissemination, gut microbiota–derived metabolites, oral microbial input, and bacterial extracellular vesicles. We further examine how these signals may interact with the BrM tumor microenvironment by influencing tumor-cell stress adaptation, myeloid inflammatory niches, antigen-presentation pathways, vascular-barrier remodeling, and metabolic reprogramming. Particular attention is given to the emerging gut–brain–metastasis axis and to cancer-type-specific contexts in breast cancer, lung cancer, and melanoma brain metastases. From a translational perspective, bacteria-related signals in BrM may eventually contribute to biomarker development, patient stratification, and therapeutic modulation of the microbe–host axis. Nevertheless, current evidence remains insufficient to conclude that BrM broadly harbor stable, active, and clinically actionable microbial communities. Future progress will require multi-source matched cohorts, longitudinal sampling, stringent low-biomass contamination control, absolute quantification, spatial validation, functional models, and explicit separation of microbial presence, viability, and causality. A rigorous evidence-based approach will be essential for moving this field from intriguing associations toward biologically interpretable and clinically meaningful applications.

## 1 Introduction

Brain metastases (BrM) are among the most common intracranial malignant tumors in adults. They frequently arise from extracranial solid tumors such as lung cancer, breast cancer, and melanoma, and are closely associated with neurological impairment, reduced quality of life, treatment failure, and poor prognosis (Nayak et al., 2012; Schreurs et al., 2025). Despite continued advances in surgical resection, stereotactic radiotherapy, whole-brain radiotherapy, targeted therapy, and immunotherapy, the overall prognosis of patients with BrM remains limited. The brain-specific immune microenvironment has therefore emerged as an important research focus for understanding disease progression, immune evasion, and therapeutic resistance (Schreurs et al., 2025). Compared with extracranial metastases, BrM do not merely grow in another anatomical compartment; rather, they are embedded within a highly specialized central nervous system tumor microenvironment (TME). This niche is constrained and remodeled by the blood-brain barrier (BBB) and the blood-tumor barrier (BTB) that develops under tumor-bearing conditions (Arvanitis et al., 2020), and is jointly constituted by brain microvascular endothelial cells, pericytes, astrocytes, neurons, perivascular niches, extracellular matrix, and a distinct metabolic state (Schreurs et al., 2025; Arvanitis et al., 2020). In parallel, microglia, monocyte-derived macrophages, T cells, and other myeloid cells play central roles in the immune ecology of BrM, contributing to tumor-cell colonization, immune regulation, vascular remodeling, and therapeutic responses (Schreurs et al., 2025; You et al., 2019; Schulz and Sevenich, 2021).

In recent years, studies of intratumoral microorganisms have suggested that tumor-type-associated bacterial signals can be detected across multiple solid tumor types, with a subset of these signals showing intracellular localization within cancer cells and immune cells (Nejman et al., 2020). Breast cancer models further indicate that low-biomass intracellular microbiota within tumors may have functional relevance in promoting metastatic colonization (Fu et al., 2022). Pan-cancer analyses of metastatic lesions have also shown that microbial signals in metastases are not randomly distributed, but may be associated with tumor type, the metastatic organ niche, hypoxic state, and local immune features (Battaglia et al., 2024). However, brain tumors and brain metastatic tissues are typically low-microbial-biomass samples. In such samples, reagent contamination, environmental contamination, sample collection and processing procedures, sequencing batch effects, biases in host-sequence depletion, and database misannotation may all generate false-positive results (Salter et al., 2014; Eisenhofer et al., 2019; Dohlman et al., 2021; Fierer et al., 2025). Therefore, findings based solely on 16S rRNA sequencing, metagenomic sequencing, or taxonomic annotation are insufficient to establish genuine tissue-resident microbial colonization. Instead, such findings must be interpreted together with negative controls, contamination correction, spatial localization, morphological validation, host-response features, and cross-modal multi-omics evidence (Salter et al., 2014; Eisenhofer et al., 2019; Dohlman et al., 2021; Fierer et al., 2025; Morad et al., 2025).

Given the current evidentiary boundaries, BrM-associated bacterial findings are best considered at three levels. The first level comprises “bacterial signals” or “bacterial elements,” including 16S rRNA, bacterial DNA/RNA, lipopolysaccharide (LPS), peptidoglycan, lipoteichoic acid, bacterial antigenic peptides, bacterial extracellular vesicles (BEVs), and bacteria-derived metabolites (Morad et al., 2025; Kalaora et al., 2021; Naghavian et al., 2023; Chronopoulos and Kalluri, 2020; Bhanu et al., 2025). This level indicates the presence of bacteria-related molecular or structural signals in a sample, but does not imply colonization by viable bacteria. The second level is “intratumoral bacteria,” for which fluorescence *in situ* hybridization (FISH), immunohistochemistry (IHC), electron microscopy, spatial molecular imaging, or multi-omics evidence should demonstrate bacterial-like structures or bacterial markers localized to tumor cells, immune cells, or stromal cells (Nejman et al., 2020; Morad et al., 2025; Gigi et al., 2025). The third level is “intratumoral microbiota/microbiome,” a term that should be used only when evidence supports community diversity, biological activity, spatial organization, and cross-cohort reproducibility, while adequately excluding contamination (Salter et al., 2014; Eisenhofer et al., 2019; Dohlman et al., 2021; Fierer et al., 2025; Morad et al., 2025).

The central question in BrM bacterial research should not remain limited to whether bacteria are present, but should extend to where these low-abundance signals are located, where they originate, whether they are biologically active, and whether they can alter the immune, vascular, and metabolic states of the BrM TME. Existing studies of brain tumors suggest that bacteria-related signals may be associated with antimicrobial responses, immunometabolic programs, and local spatial-neighborhood features (Morad et al., 2025). Analyses of brain metastases and glioblastoma samples further indicate that related microbial signatures may depend on tumor type and brain-region location, although their stability, reproducibility, and clinical relevance still require validation in larger, multicenter cohorts with rigorous quality control (Gigi et al., 2025). In addition, bacteria-derived peptides can be presented by HLA molecules and may participate in local T-cell responses (Kalaora et al., 2021; Naghavian et al., 2023); however, direct evidence that this “bacterial antigen-HLA presentation-T-cell response” mechanism also operates in BrM remains lacking. On the other hand, gut microorganisms may influence BrM initiation through a gut-to-brain axis (Massara et al., 2025), and BEVs may act as carriers for the dissemination of distal microbial signals and the modulation of host responses (Chronopoulos and Kalluri, 2020; Bhanu et al., 2025). Accordingly, this review discusses the field from five interconnected perspectives: evidentiary boundaries, potential sources, TME remodeling, cancer-type-specific context, and translational pathways.

## 2 Spatial localization and potential sources of bacterial signals in BrM

### 2.1 Differences between bacterial signals in BrM and GBM and their mechanistic implications

Brain metastases (BrM) and glioblastoma (GBM) both arise within the central nervous system, yet they differ fundamentally in tumor origin, niche formation, barrier architecture, immune composition, and therapeutic exposure. GBM is a primary central nervous system malignancy whose tumor microenvironment (TME) is mainly composed of neoplastic glial cells, reactive astrocytes, microglia/macrophages, hypoxic and necrotic regions, abnormal vascular networks, and immunosuppressive myeloid cells (Arvanitis et al., 2020; Quail and Joyce, 2017; Schaff and Mellinghoff, 2023; Klemm et al., 2020). By contrast, BrM originate from peripheral solid tumors. Metastatic cells must sequentially undergo hematogenous dissemination, adhesion to brain microvessels, traversal of the blood-brain barrier (BBB) and blood-tumor barrier (BTB), adaptation to perivascular niches, and eventual colonization of the brain parenchyma (Schreurs et al., 2025; Arvanitis et al., 2020; McDonald et al., 2023). Consequently, bacterial signals detected in BrM may be jointly shaped by the microecology of the primary tumor, circulating dissemination, BTB permeability, immune-cell trafficking, brain-region niches, and prior treatment exposure. In GBM, by contrast, related signals may be more closely linked to intratumoral hypoxia, necrosis, local immunosuppression, and abnormal vascular architecture within the primary brain tumor (Quail and Joyce, 2017; Schaff and Mellinghoff, 2023; Klemm et al., 2020).

Available comparative evidence suggests that, although BrM and GBM occupy the same central nervous system compartment, their intratumoral bacterial signatures are not identical. In a study including 322 brain tumor samples, BrM exhibited higher bacterial richness and Shannon diversity than GBM, together with tumor-type- and brain-region-associated distribution patterns (Gigi et al., 2025). In particular, BrM located in posterior brain regions showed greater bacterial-signal diversity, suggesting that local vascular architecture, BBB/BTB integrity, tumor necrosis, metabolic state, and immune ecology may jointly shape bacterial signals in BrM (Gigi et al., 2025). Nevertheless, such spatial differences should still be interpreted as heterogeneous distributions of low-abundance bacterial signals, rather than as direct evidence of stable viable-bacterial colonization or a mature intratumoral microbiome.

Some BrM bacterial signals may enter the circulation together with primary tumor cells or tumor-associated immune cells, and may then be carried into or enriched within the local TME during brain metastatic progression. At the same time, the metastatic organ itself can further shape these signals. Microbial features in metastatic lesions may be associated with tumor type, metastatic organ niche, and local immune status (Battaglia et al., 2024). Thus, BrM bacterial signals are more plausibly understood as the joint outcome of a “primary-tumor microecology-circulating dissemination-brain vascular barrier-local TME selection” process, rather than as colonization events arising from a single source.

Importantly, differences in bacterial signals between BrM and GBM do not mean that both tumor types harbor mature, active, and readily cultivable intratumoral microbiomes. A prospective multicenter study showed that intracellular bacterial 16S rRNA and LPS signals could be detected in gliomas and BrM, with localization to tumor cells, immune cells, and stromal cells; however, routine culture did not recover stable, readily cultivable bacterial communities (Morad et al., 2025). Therefore, current comparisons between BrM and GBM should focus primarily on the spatial distribution of bacterial signals, host-cell localization, tumor-type differences, and potential functional pathways, rather than prematurely generalizing them as a unified “brain tumor microbiome.”

### 2.2 Evidence for spatial and cellular localization

**FIGURE 1:**
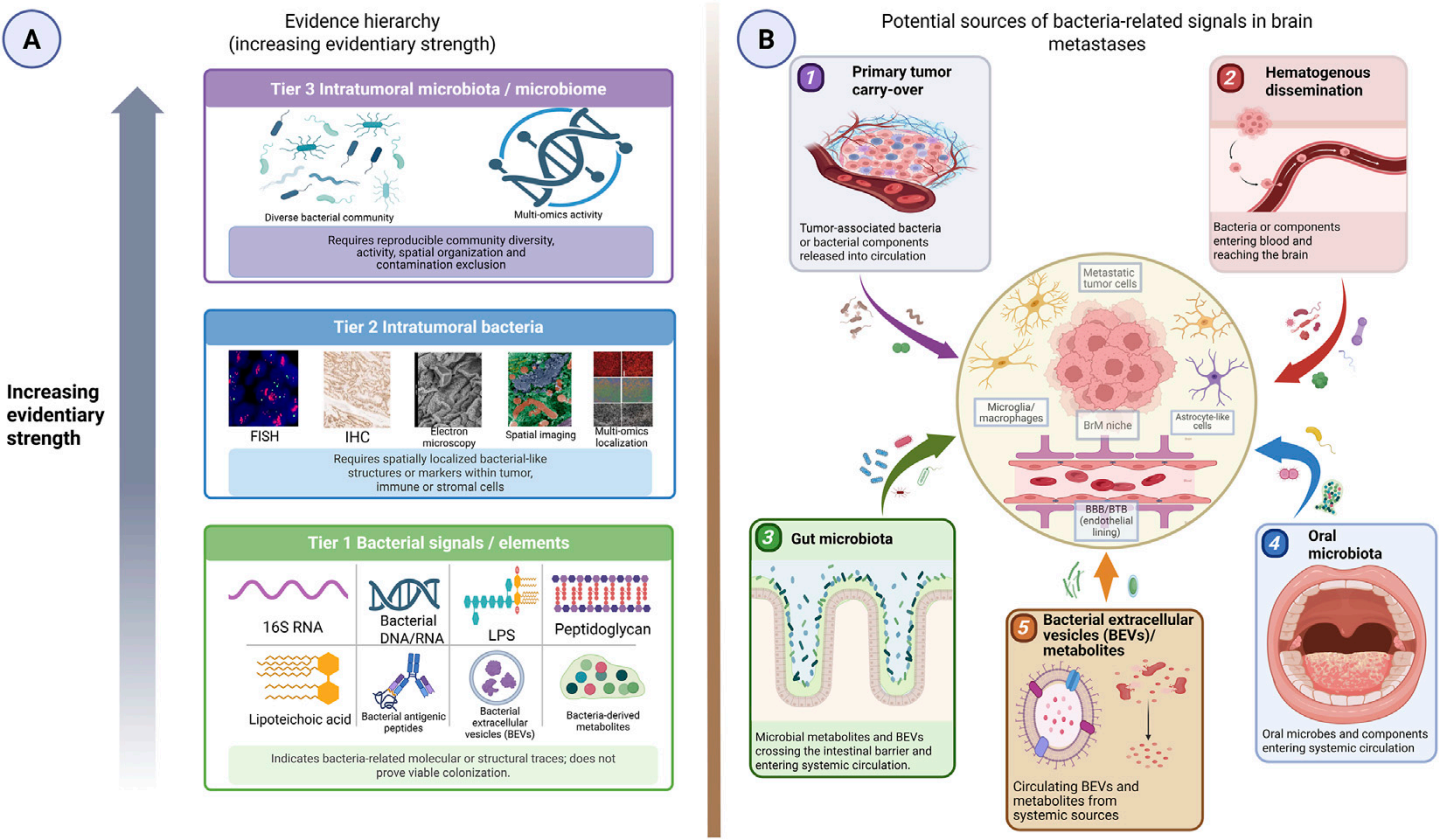
Evidence hierarchy, source models and validation strategy for bacteria-related signals in brain metastases. This figure presents a conceptual framework for interpreting bacteria-related findings in brain metastases (BrM). Panel **(A)** organizes current evidence into three ascending levels of confidence. Tier 1 refers to bacterial signals or elements, including bacterial nucleic acids, lipopolysaccharide (LPS), peptidoglycan, lipoteichoic acid, bacterial antigenic peptides, bacterial extracellular vesicles (BEVs) and microbe-derived metabolites. These signals indicate molecular or structural bacterial associations but do not establish viable colonization. Tier 2 denotes intratumoral bacteria, for which spatially resolved evidence, such as fluorescence *in situ* hybridization, immunohistochemistry, electron microscopy or spatial multi-omics, supports localization within tumour, immune or stromal compartments. Tier 3 represents an intratumoral microbiota or microbiome, a designation that should be reserved for reproducible evidence of community diversity, biological activity, spatial organization and rigorous contamination exclusion. Panel **(B)** illustrates non-mutually exclusive source models for BrM-associated bacterial signals, including primary-tumour carry-over, haematogenous dissemination, gut microbiota-derived metabolites, oral microbial inputs and circulating BEVs. These inputs may converge within the brain metastatic niche, which is shaped by the blood–brain barrier (BBB), blood–tumour barrier (BTB), tumour cells and brain-resident stromal and immune cells.

In BrM bacterial research, spatial localization evidence should be assigned a higher evidentiary weight than sequencing signals alone (Figure 1). Neither 16S rRNA amplicon sequencing nor metagenomic reads can, on their own, reliably exclude interference from DNA extraction reagents, laboratory consumables, environmental contamination, sample-to-sample cross-contamination, index hopping, host-sequence misannotation, or database bias (Salter et al., 2014; Eisenhofer et al., 2019; Fierer et al., 2025). Reliable BrM bacterial studies should therefore prioritize the integration of orthogonal evidence, including 16S rRNA FISH, LPS/peptidoglycan/EF-Tu immunostaining, electron microscopy, spatial transcriptomics, spatial proteomics, culturomics, metabolic-activity labeling, and rigorous negative controls, rather than drawing conclusions from taxonomic annotation alone.

In addition, BrM bacterial signatures are associated with tumor type, cellular localization, and anatomical location (Gigi et al., 2025). However, because current technologies have limited ability to distinguish intact viable bacteria from dead-bacterial remnants, bacterial fragments, bacterial extracellular vesicles (BEVs), or bacterial components that have been phagocytosed by immune cells, the more cautious interpretation at present is that spatially localizable bacterial signals/elements are present within the BrM TME. Whether these signals represent viable-bacterial colonization, residual bacterial structures, BEV input, or microbial components taken up by host cells remains to be established. The retraction of a cancer diagnostic study based on blood and tissue microbiome signals further underscores the need for analytical robustness and contamination control in this field (Poore et al., 2024). For low-biomass samples such as BrM, future studies should not only report negative controls and contamination-correction procedures, but should also combine spatial localization, morphological validation, viability assessment, and functional experiments to strengthen the reliability of their conclusions. Key studies and evidentiary boundaries are summarized in Table 1.

**TABLE 1:** Key studies of BrM bacterial signals and their evidentiary boundaries.

Study	Study model	Key findings	Interpretive boundary
Morad et al. (2025)	Primary and metastatic brain tumors; multiplatform analysis of human tissues	Detected low-abundance, cell-associated, and spatially heterogeneous bacterial elements associated with antimicrobial responses and spatial immune features	Supports “localizable bacterial elements/signals”; does not establish the widespread presence of stable, readily cultivable microbiota
Gigi et al. (2025)	322 GBM and BrM brain tumor samples	Microbial signatures differed between BrM and GBM in association with tumor type and brain-region location	Supports signature heterogeneity; insufficient on its own to prove viable-bacterial colonization or a mature microbiome
Massara et al. (2025)	Animal models of breast cancer BrM	The gut microbiome was associated with BrM initiation; *Alistipes* and kynurenic acid were discussed mechanistically	Provides direct preclinical support for a gut-brain-metastasis axis; human causal evidence remains lacking
Golomb et al. (2025)	BrM animal models involving antibiotic intervention and FMT	Dysbiosis was associated with BrM burden, monocyte inflammation, and dynamic changes in microglia	Suggests that distal microbial ecology can shape BrM progression, but evidence remains largely model based

### 2.3 Potential source models: primary lesions, hematogenous input, gut microbiota, and oral microbiota

The sources of bacterial signals in BrM are unlikely to follow a single route. Rather, multiple input mechanisms may converge within the brain metastatic niche. Bacterial signals may originate from primary tumors or circulating tumor cells. In colorectal cancer, *Fusobacterium* can persist within distant metastatic lesions (Bullman et al., 2017), while tumor-resident intracellular microbiota in breast cancer models can promote circulating tumor-cell survival and metastatic colonization (Fu et al., 2022). These observations support the possibility that a “primary-lesion microbial imprint” can accompany tumor dissemination to distant lesions. However, in one small study, paired primary-lesion and brain-metastasis analyses did not reveal stable or consistent microbial overlap (Morad et al., 2025). Accordingly, a carry-over model may exist in specific cancer types or patient subgroups, but it cannot be regarded as the sole source of BrM bacterial signals.

Bacterial signals may also enter the brain metastatic niche through hematogenous routes. BrM are, by nature, hematogenously disseminated disease, and the entry of bacteria-derived molecules does not necessarily require intact viable bacteria to cross an intact BBB. Bacterial DNA, LPS, peptidoglycan, bacterial proteins, BEVs, or microbial metabolites derived from primary tumors, the gut, or the oral cavity may enter regions of increased BBB/BTB permeability through the circulation, or may be internalized by monocytes, macrophages, neutrophils, and other immune cells and subsequently delivered into the local BrM TME (Arvanitis et al., 2020; Morad et al., 2025; Chronopoulos and Kalluri, 2020). Among these, BEVs can carry bacteria-derived proteins, lipids, nucleic acids, LPS, and lipoteichoic acid, and may have the capacity for distal dissemination and host-immune modulation (Chronopoulos and Kalluri, 2020). Thus, BEVs provide a plausible, although not yet directly validated, carrier model through which peripheral bacteria-derived molecules may influence the brain vascular niche and the BrM TME.

A gut-brain-metastasis axis may participate in BrM formation. In animal models of breast cancer brain metastasis, gut microorganisms can influence BrM initiation, and *Alistipes* together with the circulating metabolite kynurenic acid has been linked to cancer cell-brain vessel interactions (Massara et al., 2025). Another model study showed that antibiotic-induced gut dysbiosis increased BrM burden, whereas fecal microbiota transplantation partially reversed this effect, accompanied by changes in monocyte inflammatory responses and microglial activation dynamics (Golomb et al., 2025). These results suggest that bacteria-related effects in BrM do not necessarily require large numbers of viable bacteria to enter the brain. Instead, distal gut microbiota may shape a metastasis-permissive brain niche through peripheral immune education, circulating metabolites, vascular adhesion, and local inflammatory axes (Massara et al., 2025; Golomb et al., 2025). Nevertheless, current direct evidence still derives mainly from animal models and is insufficient to establish that human BrM risk or therapeutic response can be independently predicted by the gut microbiota.

The oral microbiota may also be a potential source of BrM bacterial signals. Some intratumoral 16 S or metagenomic signals in brain tumors show taxonomic or sequence overlap with matched oral microbiota, involving genera such as Prevotella, Capnocytophaga, *Streptococcus*, *Veillonella*, *Neisseria*, and *Bifidobacterium* (Morad et al., 2025). This observation suggests a possible connection between oral microbiota and bacterial signals in brain tumors, but taxonomic overlap alone does not establish strain-level migration, viable-bacterial colonization, or causality. Oral microorganisms may reach distant niches through periodontitis, mucosal-barrier disruption, transient bacteremia, swallowing and downstream entry into the gut, phagocytic transport by immune cells, or BEVs. Chemotherapy-associated mucositis and immunosuppression may further increase the opportunity for ectopic dissemination of oral bacteria. However, these routes remain largely mechanistic hypotheses in BrM and will require validation through strain-resolved metagenomics, spatial FISH/IHC, BEV identification, and rigorous negative controls.

Overall, available evidence supports the presence of detectable, spatially localizable bacterial signals/elements in BrM, with a degree of tumor-type and brain-region specificity. Future studies should build on stringent contamination control and integrate spatial omics, single-cell omics, culturomics, metabolomics, paired primary/metastatic cohorts, and functional intervention experiments to further distinguish bystander signals, treatment-associated changes, distal-microbiota input, and microbe-BrM interactions with genuine pathobiological significance.

## 3 How bacterial signals may remodel the BrM TME

### 3.1 Tumor cells, myeloid niches, and microbial peptide-t-cell interactions

**FIGURE 2:**
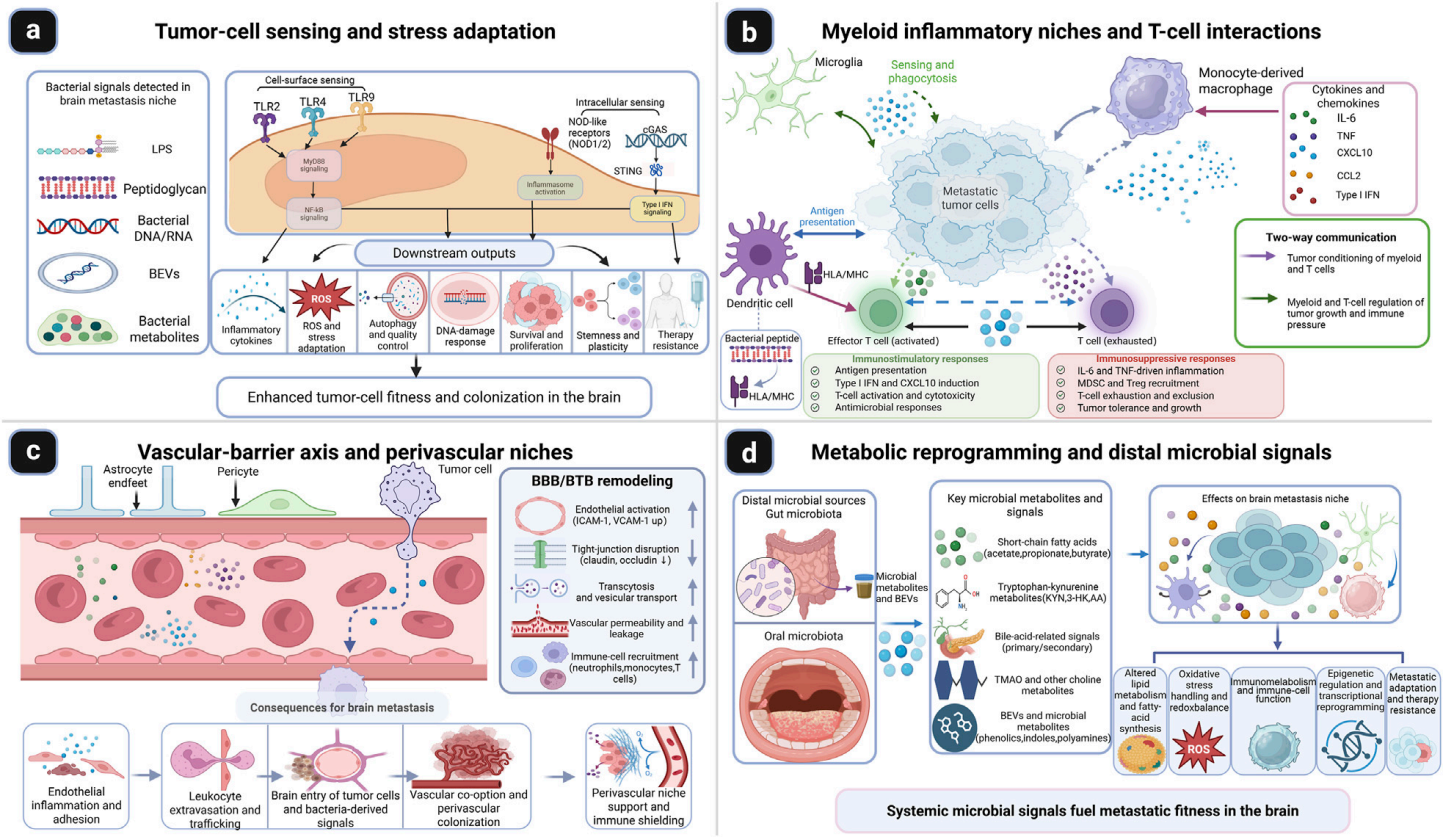
How bacteria-related signals remodel the brain metastasis tumour microenvironment. This figure summarizes plausible mechanisms through which bacteria-related signals could influence the tumour microenvironment of brain metastases. Panel **(a)** depicts tumour-cell sensing of bacterial components, including LPS, peptidoglycan, bacterial DNA or RNA, BEVs and metabolites. These cues may engage pattern-recognition pathways such as Toll-like receptors, NOD-like receptors and cGAS–STING signalling, thereby inducing NF-κB activity, type I interferon responses, inflammatory cytokines, stress-adaptation programmes, autophagy, DNA-damage responses, tumour-cell survival, phenotypic plasticity and therapy resistance. Panel **(b)** illustrates how bacterial signals may intersect with myeloid and lymphoid compartments. Microglia, macrophages and dendritic cells may sense bacteria-related products, produce inflammatory cytokines and chemokines, or process microbial peptides for HLA/MHC-mediated antigen presentation. Depending on context, this may support antimicrobial and antitumour immune activation or, alternatively, foster chronic inflammation, T-cell dysfunction and immunosuppressive niche formation. Panel **(c)** focuses on the vascular-barrier interface. Bacterial products and vesicular signals may contribute to endothelial activation, altered adhesion-molecule expression, transcytosis, focal BBB/BTB permeability, immune-cell recruitment and perivascular colonization, thereby influencing early metastatic arrest and local niche establishment. Panel **(d)** places these events within a broader metabolic framework. Distal microbial metabolites, including short-chain fatty acids, tryptophan–kynurenine products, bile-acid-related signals, trimethylamine N-oxide and BEV-associated cargoes, may modulate tumour-cell metabolism, oxidative-stress handling, immunometabolic states and brain-specific metastatic adaptation. Collectively, the figure emphasizes that bacterial signals may reshape immune, vascular and metabolic features of BrM, although direct causality remains incompletely established. BBB, blood–brain barrier; BEV, bacterial extracellular vesicle; BrM, brain metastasis; BTB, blood–tumour barrier; cGAS–STING, cyclic GMP–AMP synthase–stimulator of interferon genes; HLA, human leukocyte antigen; MHC, major histocompatibility complex.

After stringent exclusion of contamination in low-biomass samples and completion of spatial-localization validation, bacterial signals detected in BrM may be more than passive bystanders (Figure 2). They may be potentially linked to tumor-cell adaptation within metastatic brain lesions, the formation of myeloid inflammatory niches, and local antigen-presentation processes (Fierer et al., 2025; Morad et al., 2025). Bacterial 16S rRNA and LPS signals have been detected in metastatic brain tumors, and 16S-high regions in BrM were associated with upregulation of TLR9, MyD88, NF-kB/IRF-related pathways, antimicrobial responses, neutrophil chemotaxis, and antigen-presentation-related molecules (Morad et al., 2025). This pattern suggests that BrM bacterial signals are more likely associated with specific spatial niches than uniformly distributed throughout the entire tumor tissue (Fierer et al., 2025; Morad et al., 2025).

Bacterial elements may contribute to BrM colonization by influencing tumor-cell stress-adaptation programs. After entering the central nervous system, brain metastatic cells must withstand multiple selective pressures, including hemodynamic shear stress, BBB/BTB constraints, oxidative stress, nutrient limitation, astrocyte responses, and local immune clearance. Studies of peripheral solid tumors have shown that intratumoral bacteria can exhibit tumor-type specificity (Nejman et al., 2020) and are associated with immune-cell recruitment, epithelial-cell states, cell migration, and local functional heterogeneity (Galeano Niño et al., 2022). Tumor-resident intracellular bacteria can also enhance the tolerance of circulating tumor cells to fluid shear stress through remodeling of the actin cytoskeleton, thereby promoting distant metastatic colonization (Fu et al., 2022). These findings do not directly prove that identical mechanisms operate in BrM, but they provide testable biological grounds for the possibility that bacterial elements modulate cytoskeletal dynamics, adhesion and migration, stress survival, and colonization adaptation in brain metastatic cells (Nejman et al., 2020; Fu et al., 2022; Galeano Niño et al., 2022; Cao et al., 2024).

Myeloid cells may represent a key host node through which BrM bacterial signals influence the TME. The BrM microenvironment contains brain-resident microglia, peripheral monocyte-derived macrophages, border-associated macrophages, myeloid-derived suppressor cells (MDSCs), neutrophils, dendritic cells, T cells, B cells, and other immune constituents. Their proportions, spatial distribution, and functional states are jointly influenced by primary tumor type, lesion stage, brain-region location, and therapeutic exposure (Schreurs et al., 2025; Karimi et al., 2023; Chen et al., 2025). Single-cell spatial immune atlases further show that the immune composition of BrM differs substantially according to cancer type of origin (Karimi et al., 2023). At the same time, myeloid infiltration is a prominent feature of the brain metastatic microenvironment, and myeloid cells play central roles in immunosuppression, angiogenesis, tissue remodeling, and therapeutic responses (Schreurs et al., 2025; Chen et al., 2025).

Mechanistically, bacterial DNA, LPS, peptidoglycan, lipoteichoic acid, bacterial extracellular vesicles, and related components can be recognized by pattern-recognition receptors such as TLRs, NOD-like receptors, and cGAS-STING, thereby inducing NF-kB, MAPK, IRF/type I IFN, IL-1β, TNF-α, and CXCL/CCL chemokine networks (Takeuchi and Akira, 2010; Kawai and Akira, 2011; Liu et al., 2021; Johnston et al., 2021). This framework is consistent with the TLR9/MyD88/NF-kB/IRF activation, neutrophil chemotaxis, and antigen-presentation-related molecular upregulation observed in BrM 16S-high regions (Morad et al., 2025). Thus, bacterial signals in BrM may mark, and perhaps help shape, an antimicrobial-myeloid inflammatory niche. However, it remains unresolved whether these signals are drivers, concomitant phenomena, or niche features that arise secondarily after treatment and tissue injury (Morad et al., 2025; Takeuchi and Akira, 2010; Kawai and Akira, 2011; Liu et al., 2021; Johnston et al., 2021).

Notably, myeloid responses associated with bacterial signals are highly context dependent. Transient or moderate innate immune activation may enhance dendritic-cell maturation, antigen presentation, and antitumor T-cell responses. By contrast, in the setting of persistent tumor growth, hypoxic necrosis, aberrant BTB permeability, radiation injury, and chronic antigenic stimulation, prolonged low-level microbial stimulation may shift myeloid cells toward tissue-repair-like, proangiogenic, or immunosuppressive states, characterized by upregulation of ARG1, IL-10, TGF-β, PD-L1, ROS, VEGF, and CXCL/CCL chemokine axes (Schreurs et al., 2025; Chen et al., 2025; Takeuchi and Akira, 2010; Kawai and Akira, 2011; Liu et al., 2021; Johnston et al., 2021; Ma et al., 2023). This concept is consistent with studies of chronic inflammatory microenvironments in BrM. For example, sustained low-level type I IFN responses in reactive astrocytes can enhance recruitment of monocytic myeloid cells through the CCL2-CCR2 axis, thereby promoting progression of breast cancer and melanoma brain metastases (Ma et al., 2023). In addition, CNS-native myeloid cells can establish an immunosuppressive brain metastatic niche through CXCL10-related pathways (Guldner et al., 2020). Accordingly, BrM bacterial signals should not be simplistically categorized as either “pro-inflammatory” or “antitumor”; their significance is more likely to depend on host-cell type, spatial location, lesion stage, barrier state, and treatment timing.

Another potentially important mechanism is the microbial peptide-HLA-T-cell axis. In GBM, HLA immunopeptidomics has shown that HLA molecules in GBM tissues and tumor cell lines can present bacteria-specific peptides, and some HLA-II-bound bacterial peptides can be recognized by tumor-infiltrating lymphocytes, albeit weakly (Naghavian et al., 2023). In melanoma metastases, HLA-I/HLA-II-bound peptides originating from 41 bacterial species have been identified, and bacteria-derived peptides were shown to be presented by tumor cells and to induce immune responses (Kalaora et al., 2021). These studies offer important mechanistic insight for BrM: if bacterial elements in BrM are taken up by tumor cells, macrophages, dendritic cells, or B cells and subsequently processed and presented through HLA-I/HLA-II pathways, they may influence local T-cell infiltration, the TCR repertoire, T-cell exhaustion, antigen spreading, and immune-checkpoint-inhibitor responses (Kalaora et al., 2021; Naghavian et al., 2023).

Nevertheless, direct evidence from BrM remains lacking for HLA immunopeptidomics, TCR clonal tracking, and functional T-cell recognition. Therefore, microbial peptide-T-cell interactions in BrM should currently be regarded as a highly plausible but still unvalidated hypothesis. Future studies are needed to define their specific roles in antitumor immunity, immunosuppression, and therapeutic responses.

### 3.2 The vascular-barrier axis and perivascular niches

The vascular-barrier interface is one of the key entry points through which bacteria-related signals may influence the brain metastasis tumor microenvironment (BrM TME). BrM formation does not simply begin within the brain parenchyma. Rather, it starts with mechanical arrest of circulating tumor cells in the brain microvascular bed, endothelial adhesion, transendothelial migration, and subsequent survival and expansion within perivascular niches. Real-time intravital imaging studies have shown that brain metastasis formation involves sequential steps including tumor-cell arrest at vascular bifurcations, early extravasation, sustained contact with microvessels, and perivascular expansion. In parallel, metastasis-initiating cells can gain a survival advantage by adhering to pre-existing brain microvessels through vascular co-option, underscoring the perivascular niche as a core structural basis for early BrM colonization (Kienast et al., 2010; Valiente et al., 2014; García-Gómez and Valiente, 2020).

During BrM progression, the physiological blood-brain barrier (BBB) does not simply become fully open; instead, it is transformed into a blood-tumor barrier (BTB) with marked structural and functional heterogeneity. The BTB may exhibit focal increases in permeability, heterogeneous perfusion, partial preservation of tight-junction structures, altered relationships among pericytes and astrocytic endfeet, and persistent active efflux mediated by transporters such as P-glycoprotein and BCRP. This spatial heterogeneity means that the degree and distribution of entry into BrM by drugs, immune cells, circulating metabolites, and potentially microbe-derived components are highly uneven (Arvanitis et al., 2020; Lockman et al., 2010; Adkins et al., 2013). Therefore, when bacterial signals are detected in BrM, a more plausible interpretation is not that large numbers of viable bacteria have crossed an intact BBB, but rather that these signals are preferentially enriched in regions characterized by local BTB disruption, endothelial inflammation, vascular remodeling, immune-cell infiltration, and active tumor-cell extravasation. Bacteria-derived molecules may indirectly alter the vascular colonization window of BrM by inducing inflammatory endothelial states. LPS, peptidoglycan, bacterial DNA/RNA, and bacterial extracellular vesicles (BEVs) can all act as microbe-associated molecular patterns (MAMPs) and be recognized by brain endothelial cells, microglia, monocytes/macrophages, and neutrophils. Among these, the LPS-TLR4 axis is a relatively well-supported endothelial inflammatory pathway that can activate NF-kB, MAPK, and related signaling, and induce upregulation of TNF-α, IL-1β, CXCL-family chemokines, ROS, and adhesion molecules such as ICAM-1, VCAM-1, and E-selectin (Dauphinee and Karsan, 2006; Dayang et al., 2019). LPS and peripheral inflammation can compromise BBB function through disruption of tight junctions, release of inflammatory mediators, oxidative stress, matrix metalloproteinase activation, and altered transcellular transport (Peng et al., 2021; Galea, 2021). In BrM, these changes may shift the vascular endothelium from a restrictive barrier state toward a more adhesive, chemotactic, and leaky inflammatory endothelial state, thereby facilitating the arrest, extravasation, and perivascular survival of circulating tumor cells. However, the specific roles of TLR9, NOD1/2, cGAS-STING, and related pathways in vascular colonization of BrM remain primarily mechanistic hypotheses and require direct experimental validation.

Tumor-derived extracellular vesicles (EVs) have been shown to cross an intact BBB via transcytosis and to induce prometastatic changes in endothelial cells, astrocytes, and microglia (Morad et al., 2019). This finding indicates that the BBB/BTB is not a static physical barrier, but a biological interface that can be dynamically remodeled by circulating vesicular signals. EVs may carry a range of active cargoes, including LPS, lipids, proteins, DNA/RNA, and metabolites, and may interact with host immunity and the tumor microenvironment (Chronopoulos and Kalluri, 2020). Some EVs appear capable of crossing the BBB, yet their origins, dose dependence, target-cell selectivity, and mechanisms of BBB traversal remain incompletely defined. In particular, evidence for direct cerebrovascular effects of EVs derived from the human gut microbiota remains limited (Kaisanlahti et al., 2023). Therefore, in BrM, EVs or host EVs induced by bacterial signals may participate in tumor-cell vascular colonization and perivascular niche formation by enhancing endothelial inflammation, altering vesicle uptake and transcytosis, upregulating adhesion molecules, and aggravating focal BTB leakiness. However, this hypothesis still requires validation through spatial omics, colocalization of BEV markers, germ-free or antibiotic-intervention models, and endothelial-specific pathway-blockade experiments.

### 3.3 Metabolic reprogramming: distal microbial signals and brain metastatic adaptation

After metastatic cells enter the central nervous system, they do not simply retain the metabolic state of the primary lesion or extracranial metastases. Instead, they must re-establish survival advantages within the nutrient, redox, and neural-signaling environment unique to brain tissue. The brain microenvironment is characterized by high glucose consumption, distinctive lipid availability, abundance of amino acids and neurotransmitters, persistent oxidative stress, and complex perivascular nutrient niches. Accordingly, BrM cells often adapt to brain colonization and expansion by regulating glucose utilization, lipid synthesis and oxidation, amino-acid metabolism, mitochondrial function, and antioxidant programs (Boire et al., 2020; Achrol et al., 2019; Parida and Malladi, 2025; Ferraro et al., 2021; Xie et al., 2025). For example, breast cancer brain metastatic cells may exhibit increased dependence on fatty-acid synthesis, while GABA metabolic reprogramming and the FOXA2/ABAT/GABA axis in NSCLC brain metastatic cells have been shown to promote intracranial metastatic adaptation and astrocyte interactions (Ferraro et al., 2021; Xie et al., 2025). At the same time, the transition from BBB to BTB does not create a uniformly open barrier, but rather a structurally heterogeneous state marked by variable perfusion, permeability, active efflux, and drug-delivery efficiency. As a result, metabolic adaptation becomes tightly linked to vascular-barrier function, drug penetration, and local immune ecology (Arvanitis et al., 2020; Sprowls et al., 2019).

Bacteria-related signals may therefore participate in BrM metabolic remodeling through both local and distal routes. At the local level, recent studies have detected bacterial elements such as bacterial 16S rRNA and LPS in glioma and BrM samples, and have identified spatial associations between these signals and antimicrobial responses, immune-regulatory features, and metabolism-related expression patterns (Nejman et al., 2020; Morad et al., 2025). If future studies confirm that bacterial elements in BrM have genuine localization and metabolic activity, they may influence immunometabolic crosstalk among tumor cells, endothelial cells, astrocytes, and microglia/macrophages through pathways involving TLR/NF-kB, AHR, redox balance, lipid metabolism, amino-acid metabolism, and myeloid-cell function (Nejman et al., 2020; Morad et al., 2025; Massara et al., 2025; Stone and Williams, 2023). The most BrM-specific direct evidence currently comes from studies of gut microbiota regulation of the tryptophan-kynurenine-kynurenic acid axis. Germ-free conditions or antibiotic treatment can delay BrM initiation; in parallel, *Alistipes* abundance changes during antibiotic treatment and BrM progression, while the circulating metabolite kynurenic acid shows differential abundance and can influence cancer cell-brain vessel interactions in functional assays (Massara et al., 2025). The significance of this finding lies in extending the concept that “bacteria influence BrM” from local colonization to distal metabolic regulation: peripheral microbiota do not necessarily need to enter brain metastatic lesions, but may alter brain vascular adhesion, endothelial-barrier state, perivascular niches, and early colonization efficiency through circulating metabolites (Massara et al., 2025). In addition, the tryptophan-kynurenine pathway is itself a major axis of tumor immunometabolism. Kynurenine and related metabolites can regulate T-cell differentiation, the Treg-macrophage immunosuppressive axis, and responses to immune checkpoint inhibitors through receptors such as AHR. Thus, this pathway may simultaneously connect three dimensions: microbial metabolism, brain vascular interactions, and immunosuppression (Stone and Williams, 2023; Campesato et al., 2020).

Beyond tryptophan metabolism, microbe-associated metabolites such as short-chain fatty acids (SCFAs), bile acids, and trimethylamine N-oxide (TMAO) also provide important candidate mechanisms for understanding the BrM TME. SCFAs can regulate microglial maturation and function and influence effector and regulatory T-cell differentiation. Bile-acid metabolism can alter tumor immune surveillance through NKT cells, CD8^+^ T cells, or Treg-related pathways. TMAO has been reported to enhance antitumor immunity and improve responses to anti-PD-1 treatment (Erny et al., 2015; Furusawa et al., 2013; Park et al., 2015; Ma et al., 2018; Wang et al., 2022). These observations suggest that distal microbiota-derived metabolites may indirectly influence BrM ecology by modulating microglial homeostasis, myeloid-cell metabolism, vascular inflammation, T-cell function, and immunotherapy sensitivity. However, because BrM are defined by unique barrier architecture, intracranial immune constraints, and a distinctive neurometabolic background, the roles of these metabolites in peripheral tumors cannot be directly equated with their functions in brain metastases. Further validation is needed through matched patient samples, circulating metabolomics, stool/salivary microbiota analysis, spatial omics, and *in vivo* BrM models.

Bacterial metabolism may also affect therapeutic responses in BrM. Certain intratumoral Gammaproteobacteria can metabolize and inactivate gemcitabine through a long-form cytidine deaminase, thereby mediating chemoresistance (Geller et al., 2017). Although this evidence primarily derives from pancreatic and colorectal cancers and cannot yet be directly extrapolated to BrM, it suggests that microbial signals may not only alter tumor ecology but may also directly participate in drug metabolism. In BrM, the efficacy of chemotherapy, targeted therapy, radiotherapy, and immunotherapy is simultaneously influenced by BTB delivery efficiency, local hypoxia and oxidative stress, DNA damage repair, antigen presentation, and immunosuppressive ecology (Arvanitis et al., 2020; Sprowls et al., 2019; Lei et al., 2025). Therefore, if future studies establish that BrM contain genuine, spatially localized bacterial elements with transcriptional or metabolic activity, it will be important to further evaluate whether they participate in drug activation or inactivation, regulation of post-radiation oxidative stress, altered antigen presentation, and shaping of immune-checkpoint-inhibitor responses.

## 4 Cancer-type-specific context

Brain metastases (BrM) arising from different primary tumors should not be collapsed into a single concept of a “brain metastasis microbiome.” The detection and interpretation of BrM bacterial signals should instead integrate four dimensions: primary cancer type, molecular subtype, therapeutic exposure, and the intracranial niche. This approach is supported by two major considerations. First, the human solid-tumor microbiome exhibits clear tumor-type specificity, and the composition and localization of tumor-associated bacteria differ across cancer types (Nejman et al., 2020). Second, BrM themselves do not constitute a single disease entity. Distinct primary tumor types and molecular subtypes differ markedly in their intracranial vascular interfaces, immune infiltration, myeloid-cell states, glial responses, and tumor-cell functional programs (Gonzalez et al., 2022; Xing et al., 2025).

In clinical studies, radiotherapy, immune checkpoint inhibitors (ICIs), HER2/EGFR/ALK/BRAF-MEK targeted therapies, endocrine therapy, glucocorticoids, proton pump inhibitors, and antibiotics should be treated as key interpretive variables and potential confounders in BrM bacterial-signal research. Current evidence more strongly supports the view that antibiotics, proton pump inhibitors, and glucocorticoids can alter the tumor immune background by influencing gut microbiota, systemic immunity, and ICI efficacy (Routy et al., 2018; Lopes et al., 2023; Jessurun et al., 2021). By contrast, the direct effects of HER2, EGFR, ALK, BRAF-MEK targeted therapies and endocrine therapy on BrM bacterial signals remain mechanistically insufficiently established, and these variables are more appropriately handled as factors for clinical stratification and statistical adjustment. Meanwhile, the transition from BBB to BTB does not imply complete barrier opening. BTB permeability and drug delivery remain highly heterogeneous in BrM, which may also influence therapeutic exposure and the local microenvironment (Lockman et al., 2010).

### 4.1 Breast cancer brain metastases

Breast cancer BrM currently represent one of the more tractable models for investigating interactions between bacterial signals and the brain metastatic microenvironment. A major advantage is that CNS metastasis in breast cancer has a well-established clinical stratification framework: patients with HER2-positive and triple-negative breast cancer have higher risks of CNS metastasis, and molecular subtypes differ substantially in the incidence, timing, and prognosis of CNS involvement (Darlix et al., 2019). Accordingly, breast cancer BrM microbiome studies should not simply compare the presence or absence of brain metastasis, but should also incorporate HR/HER2/TNBC status, prior systemic therapy, cranial irradiation, glucocorticoids, antibiotics, and proton pump inhibitors.

Mechanistically, tumor-resident intracellular microbiota in breast cancer models can accompany circulating tumor cells and promote metastatic colonization by remodeling the actin cytoskeleton and increasing tumor-cell resistance to fluid shear stress (Fu et al., 2022). Studies using germ-free conditions and antibiotic-treated breast cancer BrM models further show that the gut microbiota can promote BrM initiation. In the same context, *Alistipes* changes differentially during antibiotic treatment and BrM progression, and the circulating metabolite kynurenic acid is associated with tumor cell-brain vessel interactions (Massara et al., 2025), providing more direct evidence in breast cancer BrM. These results suggest the existence of a gut-brain-metastasis axis in breast cancer BrM, linking gut microbiota, circulating metabolites, the brain vascular interface, and brain metastatic initiation. However, the field remains at an early stage, and the causal direction among Alistipes, kynurenic acid, and BrM initiation, the key target cells involved, and reproducibility in human BrM samples all require further validation.

Bacterial signals in breast cancer BrM must also be interpreted in the context of molecular subtype and intracranial spatial niches. Single-cell and spatial analyses have shown that TNBC and HER2-positive breast cancer exhibit different structural patterns during early brain colonization. TNBC is more likely to form perivascular sheaths and to establish more diffuse contact with astrocytes and microglia, whereas HER2-positive BrM more often form compact spheroids and displace some stromal cells toward the tumor periphery (Gan et al., 2024). These subtype-dependent spatial architectural differences suggest that, even within breast cancer BrM, bacterial signals may have different functional consequences according to HR/HER2/TNBC status, perivascular niche architecture, glial responses, and modes of myeloid-cell remodeling.

Studies exemplified by HER2CLIMB indicate that modern CNS-active therapies have substantially altered the intracranial disease-control and survival landscape of HER2-positive breast cancer BrM (Lin et al., 2020). Therefore, HER2 status, anti-HER2 treatment exposure, prior radiotherapy, glucocorticoid use, antibiotic use, proton pump inhibitor use, and sampling time point should all be treated as necessary covariates when interpreting bacterial signals in breast cancer BrM. Existing therapeutic studies are more appropriately used to support clinical stratification and confounder adjustment, rather than as direct evidence that anti-HER2 therapy alters BrM bacterial signals. In summary, microbiome-related studies of breast cancer BrM should avoid treating all breast cancer brain metastases as a single population, and even more so should avoid merging them with BrM arising from lung cancer, melanoma, renal cancer, or gastrointestinal tumors into a single “brain metastasis microbiome.”

### 4.2 Lung cancer brain metastases

Lung cancer is one of the most common primary tumor sources of brain metastases (BrM) (Achrol et al., 2019). However, bacterial signals in lung cancer BrM should be interpreted in the context of the primary pulmonary niche, the respiratory microecology, smoking/COPD background, molecular driver events, and therapeutic exposure. The lung is not microbiologically sterile. Existing studies have shown that local pulmonary commensals and lower-airway dysbiosis can participate in lung-cancer development and progression by modulating inflammatory responses, IL-17-related pathways, gamma-delta T cells, and myeloid immune states (Jin et al., 2019; Tsay et al., 2021). For example, in lung adenocarcinoma models, local commensal bacteria activate lung-resident gamma-delta T cells and promote tumor development (Jin et al., 2019), while lower-airway dysbiotic signatures enriched for oral commensals are associated with advanced lung cancer, activation of inflammatory pathways, and poor prognosis (Tsay et al., 2021). Therefore, bacterial signals in lung cancer BrM should be analyzed together with the primary lung lesion, oropharyngeal-lower-airway microbial migration, and the local pulmonary immune state.

Clinical interpretation of lung cancer BrM should also incorporate driver mutations and CNS-active targeted therapy. EGFR mutations, ALK rearrangements, and their corresponding targeted therapies alter the natural history of disease, the sampling window, and tumor immune ecology. Osimertinib, alectinib, and lorlatinib have demonstrated relatively well-established intracranial activity (Gainor et al., 2016; Reungwetwattana et al., 2018; Peters et al., 2017; Solomon et al., 2024). Accordingly, in comparisons of bacterial signals in lung cancer BrM, failure to distinguish driver mutation status, treatment exposure, and pre-versus post-treatment sampling time points may easily result in molecular subtype- and treatment-selection effects being misinterpreted as microbiome-related differences.

Immunotherapy and concomitant medication exposure are likewise key confounders in microbiome research on lung cancer BrM. Pembrolizumab has shown intracranial activity in subsets of patients with untreated or progressing melanoma/NSCLC BrM, and subsequent NSCLC BrM cohorts also suggest CNS efficacy in selected patients with PD-L1-positive disease (Goldberg et al., 2016; Goldberg et al., 2020). However, concomitant medications such as antibiotics, glucocorticoids, and proton pump inhibitors can influence ICI efficacy and survival outcomes by altering the gut microbiota, systemic immune activity, inflammatory status, and baseline patient prognosis (Routy et al., 2018; Derosa et al., 2018; Arbour et al., 2018; Dar et al., 2022). This issue is particularly important in lung cancer BrM, because patients frequently receive glucocorticoids for cerebral edema, radiation-associated reactions, or neurological symptoms, and may receive antibiotics for infection, underlying pulmonary disease, or peritreatment management. Recent lung cancer BrM-related studies further suggest that fecal metagenomics, metabolomics, and specific gut-microbiota features may be associated with the occurrence, detection, or radiotherapy response of NSCLC/SCLC brain metastases (Liu et al., 2025; Liang et al., 2025; Han et al., 2025). Yet these studies are largely based on peripheral stool samples, 16S rRNA sequencing, metagenomics, or metabolomics, and mainly provide correlational and candidate-biomarker evidence. They do not directly establish the source, colonization state, or causal function of bacterial signals within brain metastatic lesions.

Future microbiome research in lung cancer BrM should therefore adopt prospective, multi-niche, and multi-omics-integrated study designs that systematically document primary cancer type, histology, driver mutations, CNS-active targeted therapy, ICIs, radiotherapy, antibiotics, glucocorticoids, proton pump inhibitors, smoking/COPD status, and matched oral, respiratory, gut, and tumor-tissue samples.

### 4.3 Melanoma brain metastases

Studies of advanced melanoma anti-PD-1/ICI cohorts and microbiome interventions have shown that gut-microbiota diversity, community composition, and specific functional states are associated with responses to anti-PD-1 therapy; gut dysbiosis induced by antibiotic exposure may weaken ICI efficacy; higher dietary fiber intake is associated with better immunotherapy outcomes, whereas nonprescription probiotic use should not be simplistically regarded as a strategy to enhance ICI efficacy; and two early studies combining fecal microbiota transplantation (FMT) with anti-PD-1 therapy suggest that microbiota remodeling may restore or enhance antitumor immune responses in some patients with PD-1-refractory melanoma (Routy et al., 2018; Gopalakrishnan et al., 2018; Matson et al., 2018; Spencer et al., 2021; Baruch et al., 2021; Davar et al., 2021). In addition, the microbiota-derived metabolite inosine can enhance checkpoint blockade in experimental models, providing a mechanistic clue for a “microbial metabolite-T-cell immunity-ICI response” axis (Mager et al., 2020). Thus, the microbiota is indeed an important biological variable influencing melanoma immunotherapy responses. However, these observations are derived mainly from systemic advanced melanoma or non-brain-metastatic samples and should not be directly equated with MBM-specific microbiome evidence.

Intratumoral microbiome and immunopeptidomic studies further extend the mechanistic boundaries of this issue. Bacteria-derived HLA-I- and HLA-II-bound peptides have been identified in melanoma metastases, and some bacterial peptides can be recognized by T cells, suggesting that intratumoral bacterial elements may enter antigen-presentation and immune-recognition pathways (Kalaora et al., 2021). These findings support the hypothesis that intratumoral bacterial components may participate in local immune regulation. However, their relevance to BrM requires cautious interpretation: existing studies have not systematically demonstrated a stable, reproducible bacterial signature within BrM that has predictive value for intracranial therapeutic efficacy.

The clinical treatment context of melanoma brain metastases (MBM) also substantially influences the interpretation of bacterial signals. Combination immunotherapy has relatively clear intracranial activity in untreated, asymptomatic MBM patients who are not dependent on glucocorticoids, whereas patients with symptomatic disease or glucocorticoid requirements generally derive less benefit (Tawbi et al., 2018; Tawbi et al., 2021; Long et al., 2018; Long et al., 2025). Targeted combination therapy for BRAF^V600-mutant MBM can also yield high initial intracranial response rates (Davies et al., 2017). Accordingly, future analyses of MBM bacterial signals should at minimum stratify for ICIs, BRAF/MEK inhibitors, glucocorticoids, prior radiotherapy, and sampling time points. Multi-omics studies further show that, compared with primary cutaneous melanoma or extracranial metastases, BrM can display lower IFN-gamma scores, lower T-cell-inflamed scores, and reduced immune-cell infiltration (In et al., 2023; Weiss et al., 2021). Whether these immune-ecological differences are directly linked to bacterial signals remains to be determined.

### 4.4 Other cancer types and transferable mechanistic examples

Although cancers other than lung cancer, breast cancer, and melanoma are not major sources of brain metastases (BrM), they can still provide important mechanistic examples for how bacterial signals may participate in the metastatic cascade. Among them, colorectal cancer brain metastasis is relatively uncommon, with an incidence of approximately 0.6%–3.2%; it usually occurs in advanced disease and is often associated with lung metastasis, rectal primary tumors, and clinical factors such as RAS/KRAS mutations (Christensen et al., 2016). Thus, colorectal cancer is not an appropriate direct representative model for BrM microbiome research. Nevertheless, because of the close anatomical and biological relationship between colorectal primary lesions and the gut microbiota, colorectal cancer provides a strong mechanistic reference for “primary-niche-associated microbial carry-over,” “persistent intratumoral bacteria,” and “bacteria-promoted metastasis.”

With respect to bacterial persistence and primary-lesion-associated microbial carry-over, *Fusobacterium* and accompanying microbial communities can persist in colorectal cancer metastases and be retained in patient-derived xenograft models. Anti-anaerobic antibiotic treatment can reduce intratumoral *Fusobacterium* burden and inhibit tumor growth, suggesting that some tumor-associated bacteria are not merely contaminants or bystanders, but may accompany tumor clonal dissemination and participate in the formation of metastatic niches (Bullman et al., 2017). In terms of bacterial genotoxicity, *Escherichia coli* carrying the pks pathogenicity island can produce colibactin, and corresponding mutational signatures can be detected in human colorectal cancer genomes (Pleguezuelos-Manzano et al., 2020). Subsequent work has further shown that related mutational and insertion-deletion signatures are enriched in both normal colonic crypts and tumor samples from colorectal cancer patients and may contribute to early tumorigenesis (Chen et al., 2023). Regarding bacteria-promoted vascular adhesion and metastatic extravasation, *F. nucleatum* can enhance the adhesion of colorectal cancer cells to endothelial cells and facilitate tumor-cell extravasation and distant metastasis through activation of the ALPK1/NF-kB/ICAM1 axis (Zhang et al., 2022).

Taken together, these findings suggest that bacteria may influence the metastatic cascade in several ways: first, as persistent companion factors that accompany tumor dissemination and participate in metastatic-niche formation; second, by shaping tumor evolutionary trajectories through genotoxic effects; and third, by promoting metastatic colonization through regulation of endothelial adhesion, vascular extravasation, and local inflammation. However, these mechanisms should not be directly extrapolated as established mechanisms in BrM. BrM formation is constrained by specific features including the blood-brain barrier/blood-tumor barrier (BBB/BTB), brain vascular endothelium, pericytes, astrocytes, brain-resident myeloid cells, and the intracranial immune microenvironment (Arvanitis et al., 2020; Srinivasan et al., 2021).

A more reasonable translational direction is to test, in BrM samples, whether primary-lesion-associated bacteria or bacterial elements can enter the intracranial compartment together with tumor cells; whether bacteria-derived DNA, RNA, proteins, or antigenic peptides have clear spatial-localization evidence; whether bacterial signals are associated with endothelial adhesion, vascular permeability, immune exclusion, necrotic regions, therapeutic exposure, or intracranial and extracranial tumor burden; and whether such signals can alter intracranial colonization, tumor growth, immune infiltration, or therapeutic responses in in vivo BrM models, organoid models, or blood-brain-barrier coculture models. In other words, BrM provide a series of operable and testable research hypotheses spanning “bacterial persistence-genotoxicity-endothelial adhesion-metastatic extravasation-local immune regulation.”

## 5 Translational prospects and future directions

### 5.1 Biomarkers: from single samples to multi-source matched cohorts

The most immediate translational direction for BrM-associated bacterial signals is their development as candidate biomarkers for patient stratification, intracranial progression-risk assessment, treatment-response monitoring, and early warning of recurrence. However, current evidence is better understood as indicating “detectable, localizable, and biologically associated candidate signals,” rather than established clinical biomarkers. Intratumoral 16S signals are associated to some extent with antimicrobial responses, immunometabolic signatures, and matched oral/gut microbiota, but in clinically heterogeneous cohorts, the intratumoral 16S signal itself has not yet independently predicted patient characteristics, tumor characteristics, or clinical outcomes (Morad et al., 2025). Therefore, BrM bacterial signals should currently be positioned as candidate biomarkers with translational potential, rather than deterministic indicators that can be directly used for clinical decision-making.

From a study-design perspective, a single BrM tissue sample is insufficient to determine the source, activity, and functional significance of bacterial signals. Pan-cancer studies have suggested that different tumor types may harbor relatively specific intratumoral bacterial profiles, and that bacteria-related signals can occur within both tumor cells and immune cells (Nejman et al., 2020). Studies of metastatic cancers also show that the metastatic-lesion microbiome can be associated with tumor type, metastatic site, host transcriptional programs, and clinical variables (Battaglia et al., 2024). Accordingly, an ideal design for future BrM biomarker research would involve multi-source matched cohorts incorporating primary tumors, extracranial metastases, BrM, blood/plasma, CSF, stool, saliva, or oral swabs, with longitudinal sampling at diagnosis, surgery, before and after radiotherapy, ICIs, or targeted therapy, and at intracranial recurrence (Nejman et al., 2020; Battaglia et al., 2024; Dohlman et al., 2021; Morad et al., 2025). Key questions include whether bacterial signals in BrM share sources with the primary lesion or extracranial metastases; whether they more closely resemble distal microbiota in the oral cavity, gut, or blood circulation; and whether their dynamic changes are related to BTB status, the local immune microenvironment, therapeutic pressure, and intracranial progression (Arvanitis et al., 2020; Battaglia et al., 2024; Morad et al., 2025). Particularly in BrM research, CSF and blood-based samples may complement minimally invasive monitoring, but their interpretation must be integrated with tissue spatial localization, negative controls, and the clinical timeline to avoid misclassifying low-abundance microbial DNA from circulating sources, environmental sources, or experimental contamination as intratumoral colonization.

Robust biomarker studies must also fully document and adjust for clinical confounders. Antibiotics, proton pump inhibitors, diet, and hospitalization exposures can alter gut or oral microbiota backgrounds. Glucocorticoids, radiotherapy, ICIs, targeted therapy, and driver-gene status can reshape the tumor immune microenvironment. Surgical duration, tumor-necrosis proportion, sample-preservation method, and library-preparation batch may influence low-biomass detection results (Routy et al., 2018; Gopalakrishnan et al., 2018; Matson et al., 2018; Vich Vila et al., 2020; Imhann et al., 2016; Derosa et al., 2022). Prior ICI-microbiome studies have shown that gut-microbiota composition and antibiotic exposure are associated with the efficacy of PD-1/PD-L1 blockade, indicating that clinical interpretation of BrM bacterial signals must incorporate systemic microbiota background and therapeutic exposure (Routy et al., 2018; Gopalakrishnan et al., 2018; Matson et al., 2018; Derosa et al., 2022). Future biomarker research should also prospectively distinguish analytical validity, biological validity, and clinical utility, avoiding premature attribution of clinical decision-making significance to candidate signatures before the evidence is sufficient.

### 5.2 Minimum methodological standards: low biomass is not a technical detail, but a core issue

The greatest risk in BrM bacterial-signal research is not a lack of biological imagination, but the susceptibility of low-biomass samples to contamination, cross-contamination, batch effects, host-sequence misannotation, and database bias. Brain tissue and brain metastases are not typical high-microbial-burden environments. Therefore, bacterial DNA, RNA, or antigenic signals detected in such samples must first withstand rigorous methodological scrutiny. Contaminating DNA from extraction kits and laboratory reagents can substantially influence 16S rRNA sequencing and shotgun metagenomic results in low-biomass samples (Salter et al., 2014). Low-biomass microbiome studies should implement contamination control throughout sample collection, handling, DNA extraction, library preparation, sequencing, and bioinformatic analysis, and should adopt minimum experimental standards to reduce false-positive interpretations (Eisenhofer et al., 2019). They must also systematically report contamination control, cross-contamination identification, and contamination-removal workflows; otherwise, reliable comparison and reproducibility across studies become difficult (Fierer et al., 2025). In BrM, low biomass should therefore not be regarded as a mere technical detail, but as a core issue that determines the credibility of conclusions.

Statistical decontamination methods can serve as necessary complements, but cannot replace front-end experimental control. decontam can identify contaminant sequences using frequency-based and prevalence-based strategies based on DNA concentration gradients and frequencies of occurrence in negative controls (Davis et al., 2018). However, no bioinformatic decontamination procedure can fully compensate for systematic bias introduced by inadequate control during sampling, extraction, PCR, or library preparation, nor can it independently establish the genuine intratissue localization of bacterial signals. The Cancer Microbiome Atlas emphasizes the need to distinguish true tissue-resident microbiota from contaminants through systematic comparison and contamination identification (Dohlman et al., 2021). Reanalyses of cancer microbiome datasets suggest that host-sequence misclassification, reference-database problems, and data-processing errors can all generate substantial false positives (Gihawi et al., 2023; Ge et al., 2025). Recent methodological commentaries and consensus papers on intratumoral microbiota research further argue that microbial presence, viability, and causality should each be assigned separate evidentiary thresholds and cannot be substituted for one another by any single detection platform (Jawahar and Byrd, 2025; Duan et al., 2026). In addition, PRISM, a computational framework for low-biomass human genomic data, appears to improve the reliability of microbial-signal identification and decontamination, while emphasizing that sequencing strategy itself influences interpretation of positive and negative findings (Ghaddar et al., 2026). Nonetheless, such downstream algorithms can only improve interpretation quality; they cannot replace front-end design for sampling, extraction, library preparation, and negative controls.

BrM bacterial-signal studies should meet at least the following methodological standards. During sampling and experimentation, investigators should include air-exposure controls, operating-room environmental controls, instrument or bench-surface controls, extraction blanks, PCR-negative controls, and library-preparation negative controls, while simultaneously incorporating positive controls, mock communities, spike-in controls, or dilution-series samples to assess detection sensitivity, batch bias, and contamination background (Salter et al., 2014; Eisenhofer et al., 2019; Fierer et al., 2025; Davis et al., 2018). Different specimen types, including tissue, blood, CSF, stool, saliva, oral swabs, and respiratory samples, should be processed with physical segregation, and batch randomization should be applied during sample extraction, PCR amplification, and library preparation to minimize cross-contamination among different sources from the same patient (Eisenhofer et al., 2019; Fierer et al., 2025; Mirzayi et al., 2021). Beyond sequencing, orthogonal validation should incorporate 16S rRNA RNAscope/FISH, LPS, EF-Tu, or peptidoglycan IHC, transmission electron microscopy (TEM), spatial transcriptomics, spatial proteomics, digital spatial profiling, bacterial-activity labeling, culture-based methods, or culturomics to determine whether signals are truly localized to tumor cells, immune cells, stromal cells, or necrotic regions (Fierer et al., 2025; Morad et al., 2025; Gigi et al., 2025). During interpretation, investigators must clearly distinguish bacterial DNA, bacterial RNA, intact bacteria, dead-bacterial remnants, bacterial extracellular vesicles, extracellular contamination, and host-cell phagocytic signals, thereby avoiding direct equivalence between “molecular traces of bacterial association” and “active colonizing microbial communities” (Eisenhofer et al., 2019; Dohlman et al., 2021; Fierer et al., 2025; Morad et al., 2025).

Finally, the clinical significance of BrM bacterial signals must be interpreted within the tumor niche rather than in isolation. Future studies should jointly model bacterial signals with therapeutic timelines, lesion spatial location, tumor-necrosis proportion, BTB integrity, local hypoxia, immune-cell composition, antimicrobial/inflammatory pathways, and intracranial and extracranial disease burden (Arvanitis et al., 2020; Battaglia et al., 2024; Morad et al., 2025). Research reports should also follow microbiome reporting guidelines such as STORMS and fully disclose sample collection, storage, DNA/RNA extraction, sequencing platforms, negative/positive controls, contamination identification, host-sequence depletion, database versions, statistical models, and sensitivity-analysis procedures (Jawahar and Byrd, 2025; Duan et al., 2026; Mirzayi et al., 2021). Only when contamination control is adequate, spatial localization is clear, host responses are consistent, longitudinal dynamics are reproducible, and mechanistic experiments or intervention studies support a causal chain can BrM bacterial signals genuinely progress from “correlational These minimum standards are summarized in Table 2. findings” to “causal mechanisms” and “clinically translatable tools.”

**TABLE 2:** Minimum methodological standards for BrM bacterial-signal research.

Research component	Minimum recommendation
Sampling design	Record surgical time, lesion location, necrosis proportion, preservation method, and therapeutic exposure; prioritize matched cohorts and longitudinal sampling
Contamination control	Include air, operating-room environmental, instrument/bench-surface, extraction-blank, PCR-negative, and library-preparation negative controls, together with positive controls, mock communities, or spike-in controls
Sequencing and decontamination	Report host-sequence removal, database versions, threshold settings, and sensitivity analyses; tools such as decontam and PRISM may be used, but algorithms must not replace front-end experimental control
Spatial and cellular localization	Integrate FISH/RNAscope, IHC, TEM, spatial transcriptomics, spatial proteomics, or digital spatial profiling to define localization in tumor, immune, and stromal cells
Biological interpretation	Distinguish bacterial DNA/RNA, phagocytosed intracellular components, BEVs, dead-bacterial remnants, and active bacteria; discuss presence, viability, and causality separately
Clinical statistics	Adjust for antibiotics, proton pump inhibitors, glucocorticoids, radiotherapy, ICIs, targeted therapy, driver mutations, smoking/COPD, and related confounders
Reporting standards	Follow reporting guidelines such as STORMS and fully disclose controls, batches, statistical models, negative results, and study limitations

### 5.3 Therapeutic targets: from “eliminating bacteria” to “precisely modulating the microbe-host axis”

The therapeutic translation of bacterial signals in BrM should not be simplistically understood as “detect bacteria, then eliminate them with antibiotics.” A more appropriate objective is to precisely identify actionable taxa, PAMPs, metabolites, or host-response nodes after clarifying bacterial sources, spatial localization, host responses, and causal roles.

From a biological perspective, intratumoral or tumor-associated bacterial signals may exert bidirectional effects. Some microbial signals may enhance antitumor immunity through pattern-recognition-receptor signaling, the STING-type I IFN axis, antigen presentation, T/NK-cell activation, and tertiary lymphoid structure (TLS) formation. For example, STING signaling has been shown in certain models to enhance anti-CD47 immunotherapy responses (Shi et al., 2020). GBM studies also demonstrate that HLA molecules can present bacterial peptides that are weakly recognized by tumor-infiltrating lymphocytes, suggesting that bacterial antigens may participate in immune recognition of CNS tumors (Naghavian et al., 2023). Conversely, other bacterial signals may promote tumor progression or therapeutic resistance through chronic inflammation, ROS production, NF-kB activation, myeloid immunosuppression, neutrophil recruitment, T-cell dysfunction, metabolic reprogramming, and vascular-barrier alterations. Recent work further shows that a higher intratumoral bacterial burden is associated with neutrophil enrichment, T-cell depletion, and resistance to immune checkpoint inhibitors (Silver et al., 2026).

One strategy that merits priority validation is intervention against specific taxa, bacterial components, or host-response nodes that have been rigorously shown to promote metastasis or therapeutic resistance. The key prerequisite is not merely the detection of bacterial sequences, but demonstration that the signal has spatial localization, host interaction, functional causality, and tractability for intervention. Colorectal cancer provides important mechanistic examples: *F. nucleatum* can persist in primary and metastatic lesions and is associated with tumor growth and antibiotic responsiveness (Bullman et al., 2017); *Escherichia coli* can generate colibactin-associated genotoxic mutational signatures (Pleguezuelos-Manzano et al., 2020); and can enhance colorectal cancer-cell adhesion to endothelial cells and promote extravasation and metastasis through the ALPK1/NF-kB/ICAM1 axis (Zhang et al., 2022). These findings cannot be directly extrapolated as established mechanisms in BrM, but they provide an experimental framework for BrM research centered on “bacterial persistence-endothelial interaction-metastatic-niche formation-functional intervention validation.”

Another testable direction is to target host-response pathways induced by bacterial signals, rather than directly targeting bacteria themselves. Potential nodes include TLR4-LPS, TLR9-bacterial DNA, NOD-like receptor signaling, cGAS-STING, NF-kB, myeloid inflammation, neutrophil recruitment, tryptophan-kynurenine metabolism, endothelial activation, and BBB/BTB remodeling. Importantly, the evidence supporting these pathways in BrM is not uniform. The spatial immunological associations of LPS/16S signals are supported by brain-tumor studies (Morad et al., 2025); STING-related antitumor immunity derives mainly from non-BrM models (Shi et al., 2020); and NF-kB/ICAM1-mediated endothelial adhesion and extravasation are currently supported primarily by colorectal cancer studies (Zhang et al., 2022). Therefore, it is more appropriate to frame these pathways as host-response nodes deserving priority validation in BrM bacterial-signal research, rather than as already established BrM therapeutic targets.

Modulation of the gut microbiota and its metabolites may indirectly influence systemic immune status and intracranial antitumor responses. Multiple studies have shown that gut-microbiota composition is associated with responses to PD-1/PD-L1 therapy. Gopalakrishnan, Matson, and Routy and colleagues respectively reported differences in gut-microbiota architecture between responders and nonresponders among patients with melanoma or epithelial tumors, and antibiotic exposure may weaken immune-checkpoint-inhibitor efficacy (Routy et al., 2018; Gopalakrishnan et al., 2018; Matson et al., 2018). Fecal microbiota transplantation combined with anti-PD-1 therapy can reshape the gut microbiota and tumor immune microenvironment in some patients with PD-1-refractory melanoma and restore a degree of treatment responsiveness (Davar et al., 2021). However, this evidence mainly derives from melanoma or other solid tumors rather than BrM-specific clinical studies. Thus, in BrM, interventions involving diet, prebiotics, postbiotics, FMT, or specific metabolites are currently better positioned as preclinical or early translational research directions than as mature therapeutic recommendations.

Engineered bacteria, bacteriophages, and bacterial extracellular vesicles (BEVs) may be viewed as longer-term delivery and immunomodulatory platforms. Engineered bacteria can improve tumor targeting and therapeutic controllability through surface modification, synthetic genetic circuits, kill switches, and localized payload release (Wang et al., 2026). BEVs have also attracted attention because of their nanoscale size, engineering potential, and delivery capacity. However, in the BrM setting, such strategies remain far from clinical application and must first address CNS safety concerns, including infection risk, cerebral edema, neuroinflammation, uncontrolled immune activation, BBB/BTB delivery efficiency, horizontal gene transfer, and neurotoxicity when combined with radiotherapy or immunotherapy. Their appropriate current positioning is therefore as exploratory therapeutic platforms requiring stringent safety validation, rather than as directly translatable clinical options.

**FIGURE 3:**
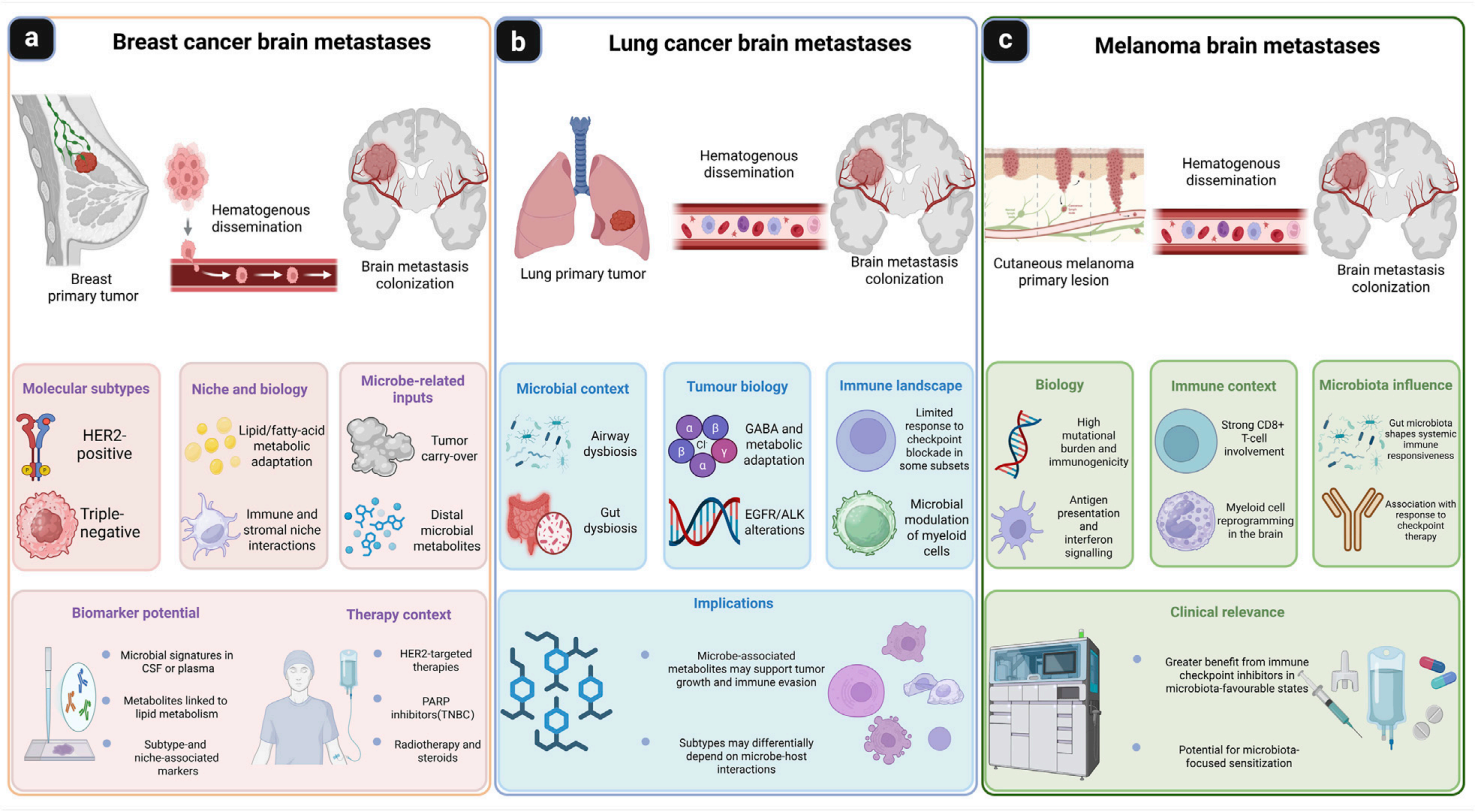
Cancer-type-specific contexts and translational roadmap for microbe–host interactions in brain metastases. This figure integrates tumour-type heterogeneity with a translational framework for studying bacteria-related signals in brain metastases. Panels **(a-c)** highlight that BrM arising from breast cancer, lung cancer and melanoma should not be interpreted as a single biological entity. In breast cancer BrM (panel **(a)**), microbial questions intersect with molecular subtype, brain-colonization architecture, lipid metabolic adaptation and therapeutic context, including HER2-directed therapy and triple-negative disease. In lung cancer BrM (panel **(b)**), relevant variables include airway and gut dysbiosis, haematogenous dissemination, molecular drivers such as EGFR or ALK alterations, brain-adaptive metabolism and distinct immune-checkpoint responses. In melanoma BrM (panel **(c)**), bacterial and microbiota-related hypotheses are especially relevant to immunogenicity, antigen presentation, T-cell activity and responsiveness to immune-checkpoint blockade. Across all three tumour types, bacterial signals may arise from primary-tumour ecosystems, distal mucosal microbiota or systemic metabolite and vesicle trafficking, but their significance must be interpreted within disease-specific biological and therapeutic contexts. Panel d proposes a translational roadmap. Progression from descriptive association to clinical application requires matched multi-source cohorts, longitudinal sampling, contamination-aware low-biomass workflows, absolute quantification, spatial multi-omics, and functional validation in organoids, brain-slice systems and *in vivo* models. These steps can support biomarker discovery, patient stratification and rational testing of interventions that modulate the microbe–host axis, including metabolite-guided strategies, microbiome-informed immunotherapy or targeted manipulation of bacterial extracellular vesicle pathways. The figure underscores that clinical translation requires explicit separation of microbial presence, viability, function and causality. ALKanaplastic lymphoma kinase, ALK, anaplastic lymphoma kinase; BEV, bacterial extracellular vesicle; BrM, brain metastasis; EGFR, epidermal growth factor receptor; HER2, human epidermal growth factor receptor 2; ICI, immune-checkpoint inhibitor.

Overall, the therapeutic significance of BrM bacterial signals lies not in proposing a generalized “antimicrobial treatment,” but in establishing a more refined framework for intervening in the microbe-host axis (Figure 3). Future studies should prioritize multi-source matched cohorts spanning primary lesions, brain metastases, blood, and stool/oral samples, together with low-biomass contamination control, spatial localization, multi-omics integration, functional experiments, and causal validation to distinguish bystander signals from driver signals. At the translational level, study endpoints should not be limited to bacterial load or taxonomic changes, but should also include intracranial progression-free survival, intracranial immune phenotypes, cerebral edema, neurotoxicity, antitumor treatment response, and microbiota ecological perturbation. Only when a specific bacterial signal is shown to be reproducible, localizable, actionable, and causally associated with clinical outcomes can the microbe-host axis in BrM genuinely move from correlational observation to a therapeutically targetable stage.

## 6 Conclusion

Future research on BrM bacterial signals should move beyond being merely “detectable” toward becoming “interpretable, verifiable, and translatable.” First, it is necessary to clarify whether bacterial signals detected in BrM represent viable bacteria, unculturable or dormant bacteria, dead bacteria, bacterial fragments, LPS or other membrane components, fragmented bacterial RNA/DNA, or molecular remnants originating from distal mucosal microbiota. Morad and colleagues detected low-abundance, cell-associated bacterial elements in primary and metastatic brain tumors using FISH, IHC, spatial molecular imaging, digital spatial profiling, sequencing, culturomics, and other approaches. However, their findings did not support the widespread presence of readily cultivable microbiota in brain tumors. Accordingly, BrM bacterial signals should not be simply equated with a stable, active, and cultivable brain-metastasis microbiome (Morad et al., 2025).

The spatial distribution, cellular attribution, and coupling of BrM bacterial signals to lesion niches must also be resolved. Future work should clarify whether these signals are primarily localized to tumor cells, myeloid cells, T/NK cells, endothelial cells, pericytes, astrocytes, or other stromal compartments, and should further determine whether they are associated with necrosis, hypoxia, perivascular regions, immune-cell infiltration, post-treatment changes, and BTB permeability. Studies in other solid tumors have already suggested that intratumoral microbiota can display nonrandom spatial distributions and may be linked to local immune and invasive niches. However, these patterns cannot be directly extrapolated to BrM and still require orthogonal validation in brain metastatic tissues through FISH/IHC, spatial transcriptomics, spatial proteomics, single-cell sequencing, and high-resolution imaging (Arvanitis et al., 2020; Nejman et al., 2020; Morad et al., 2025; Galeano Niño et al., 2022).

It is also necessary to test whether the gut-brain-metastasis axis can predict BrM risk, postoperative intracranial recurrence, and responses to ICIs or targeted therapies. Breast cancer brain metastasis models indicate that the gut microbiota can influence BrM initiation through a gut-to-brain axis, providing experimental support for the concept that distal microbial communities can regulate brain metastatic colonization (Massara et al., 2025). Antibiotic-induced dysbiosis and its partial correction by FMT likewise suggest that peripheral microbial ecology may affect BrM progression (Golomb et al., 2025). Nonetheless, these data still arise mainly from animal models and do not directly demonstrate that human BrM risk can be independently predicted by the gut microbiota. Clinical studies must further disentangle relationships among therapeutic exposure, glucocorticoids, antibiotics, proton pump inhibitors, diet, host immune status, and BrM bacterial signals.

Whether CSF, blood, and oral/stool/respiratory microbiota can be developed into noninvasive BrM biomarkers remains exploratory. Because of its anatomical proximity to CNS lesions, CSF may have particular potential in liquid biopsy approaches for intracranial tumors. Existing studies have shown that CSF ctDNA, CTCs, EVs, and protein markers may be valuable for monitoring CNS tumors and brain metastases. Future work may further explore whether microbial DNA/RNA, BEV-like microbial products, or host antimicrobial-response features can be jointly modeled with imaging, ctDNA, immunometabolic indicators, and clinical endpoints (Morad et al., 2025; Robinson et al., 2024; Diaz et al., 2024; Morganti et al., 2023). Supporting the feasibility of this broader biomarker concept, a recent study (Yu et al., 2025) showed that intratumoral microbiome features and circulating microbial DNA signatures were associated with osteosarcoma pulmonary metastasis, and further linked microbiome-derived butyrate to Wnt/β-catenin-driven metastatic progression. Although this study does not provide direct evidence for BrM, it illustrates how microbial DNA profiling and microbial metabolite analysis may be integrated into metastasis-oriented biomarker frameworks. Future BrM studies should test whether analogous microbial or microbial-metabolic signatures can be detected and validated in matched CSF, blood, stool/oral/respiratory, and tumor-tissue cohorts with rigorous contamination control.

At the current level of evidence, however, these microbial biomarkers cannot be regarded as mature clinical tools and must undergo longitudinal sampling, independent validation, and stringent contamination control before moving toward clinical translation. Overall, BrM bacterial signals define an emerging interdisciplinary field linking low-biomass tumor microbial signals, brain-specific immunometabolic ecology, vascular-barrier biology, mechanisms of tumor-cell colonization, and the gut-brain-metastasis axis. Current evidence supports the presence of low-abundance, spatially heterogeneous, cell-associated bacterial signals/elements in the BrM TME and suggests possible associations with antimicrobial responses, myeloid inflammation, immunometabolism, vascular-barrier status, and distal mucosal microbiota. However, the existing evidence remains insufficient to broadly conclude that BrM harbor stable, active, and cultivable brain-metastasis microbiomes. Truly valuable future studies should do more than report that “certain bacteria are detected in brain metastases”; they should establish source, spatial location, biological activity, host-response pathways, clinical associations, and tractability for intervention. Only when matched cohorts, stringent contamination control, absolute quantification, spatial localization, orthogonal validation, and functional models are jointly satisfied can BrM bacterial signals progress from an “interesting correlation” to the realm of biomarkers and therapeutic targets (Jawahar and Byrd, 2025; Duan et al., 2026; Ghaddar et al., 2026).
